# The Basic Empathy Scale in Chinese College Students: Adaptation and Psychometric Properties of a Revised Form

**DOI:** 10.3389/fpsyg.2021.774199

**Published:** 2021-12-01

**Authors:** Yin Chen, Guangbo Dou, Liang Chen

**Affiliations:** ^1^College of Kinesiology, Shenyang Sport University, Shenyang, China; ^2^School of Marxism, University of Science and Technology Liaoning, Anshan, China

**Keywords:** college students, revision, BES, psychometric properties, exploratory structural equation modeling

## Abstract

This study aimed to revise the Chinese version of the Basic Empathy Scale for college students. The cluster random sampling method was used to select 805 college students from two universities to conduct confirmatory factor analysis, correlation analysis, reliability analysis, and an independent samples *t*-test. The confirmatory factor analysis model illustrated that the two-factor model failed to fit the data, and the two-factor model with methodological effect was finally accepted. Therefore, the questionnaire exhibits a strong methodological effect among Chinese college students which requires further study. Emotional and cognitive empathy had a significant positive correlation with gratitude and Internet altruism behavior, which showed good convergent validity. The gender difference test revealed that the emotional empathy level of girls was significantly higher than that of boys. The revised Basic Empathy Scale showed acceptable reliability and validity.

## Introduction

Empathy is a combination of cognitive ability and emotional response, particularly the ability or tendency to perceive other people’s feelings and emotional states (Chen and Shi, 2007). Gladstein (1983) proposed two main types of empathy: cognitive and emotional. Cognitive empathy refers to an understanding of the purposes, intentions, and beliefs of others, whereas emotional empathy refers to the feelings of others’ emotional states (Chen, 2013). College students experience a critical period of transition from adolescence to adulthood. Moreover, their low empathy levels could characterize psychological problems, such as autism, alexithymia, and depression. Swart et al. (2009) found that college students with high levels of alexithymia scored poorly on first-level emotional tasks, which indicates a deficiency in their ability to understand other people’s emotions. Similarly, Deng et al. (2017) discovered that serious alexithymia indicated an obvious defect in empathy. Moreover, Zhang (2016) found that depression among college students was significantly and negatively correlated with empathy. The dynamic model of empathy (Liu et al., 2009) posits that people with low levels of empathy exhibit a low ability to deal with other people’s behavior and difficulty in understanding other people’s emotions and empathizing with them; their behavior too is less likely to be understood by others, increasing their tendency to experience interpersonal distress. It is therefore clear that empathy has an important positive effect on the cultivation of personality among college students and optimizes interpersonal relationships. Further research should be conducted on college Students’ ability to empathize.

Numerous measurement tools for empathy are available, the most common being the Hogan Empathy Scale (HES) (Hogan, 1969) to measure cognitive empathy, the Questionnaire Measure of Emotional Empathy (QMEE) (Mthrabian and Epstien, 1972) to measure emotional empathy, the Interpersonal Reactivity Index (IRI) (Davis, 1996), and the Basic Empathy Scale (BES) (Darrick and David, 2006) to measure multidimensional empathy. In addition to the two dimensions of appropriate accommodation and socializing style, HES also has sensitivity and other orientations. Therefore, HES is not a pure empathy scale, but more like a social skills scale (Johnson et al., 1983). The controversy over QMEE argues that it is related to the ability to evoke emotions in the overall environment, rather than specifically targeting human emotions (Mehrabian et al., 1988). In addition, Darrick believes that QMEE confuses empathy with sympathy and uses college students as a norm to assess the empathy level of offenders or similar groups (Darrick and David, 2006). The Interpersonal Reactivity Index (IRI), which is widely used in China, measures other non-empathic variables related to empathy, such as fantasy and personal pain, which are more similar to evaluating imagination and self-emotional control (Simon and Sally, 2004). Darrick and David (2006) introduced the BES to circumvent the shortcomings of the previous main empathy scale, which was suitable for the tenth grade. This scale has been widely used in several cultures with acceptable reliability and validity. For example, the Cronbach’s α of the French youth revision of the BES was 0.80 and of the emotional and cognitive empathy subscales were 0.77 and 0.66, respectively, with acceptable structural viability (D’Ambrosio et al., 2009). The Cronbach’s α of the Italian youth revision of the BES was 0.87 and that of the emotional and cognitive empathy subscales were 0.86 and 0.74, respectively, with good structural validity (Albiero et al., 2009). Li et al. (2011) revised the BES among the Chinese youth population and used confirmatory factor analysis to support the two-factor model with methodical effect (reverse-scoring items as the third dimension), and found that the questionnaire had an acceptable coefficient of internal consistency, with a Cronbach’s α of 0.777 for the total scale. Meanwhile, the Cronbach’s α of the cognitive and emotional empathy scales were 0.746 and 0.718, respectively. However, Carré et al. (2013) revised the BES among adult samples and found that three dimensions, namely the Cronbach’s α *of* emotional contagion (CONT), cognitive empathy (EMP), and emotional disconnection (DIS), were 0.72, 0.69, and 0.80, respectively, with good structural validity. Therefore, the psychological structure of empathy may be influenced by culture and age.

Ding and Song (2017) discovered that college Students’ ability to empathize was significantly and positively correlated with gratitude and that individuals with high gratitude were likely to exhibit strong empathy responses to other people’s unfortunate events, thereby enhancing their helping behavior. Individuals with a high sense of gratitude are likely to experience and feel others’ emotions and enhance empathic responses toward others. Furthermore, empathy has a significant and positive predictive effect on Internet altruistic behavior (Jiang et al., 2016). Internet altruistic behavior is a voluntary act that benefits others in a network situation, while helpers lack a clear selfish motive (Peng and Fan, 2005). This behavior manifests in the reminder, support, and guidance of others in cyberspace, as well as in information sharing with others, which is a positive pro-social behavior. Therefore, this study assumes that empathy is significantly and positively related to gratitude traits and Internet altruistic behavior.

Based on the presented theoretical basis and practical requirements, given that the BES cannot be applied directly to college students in China, this scale needs to be revised for Chinese college students. Therefore, the scale’s reliability and validity among college students were tested to develop a BES suitable for college students in China.

## Materials and Methods

### Participants

The study protocol was approved by the Research Ethics Committee of the University of Science and Technology Liaoning (China). The cluster stratified sampling method was used to select two universities in two Chinese cities. Eight majors (materials, chemical industry, automation, mathematics, physics, management, foreign languages, and education) were taken as clusters. The four grades were classified as stratification. Random sampling was then performed for different majors in the four grades. A total of 850 college students participated in the study, of which 805 were included in the sample, with an age range of 17–23 years and an average age of 20.46 years (*SD* = 1.45). A total of 522 (64.8%) boys and 283 girls (35.2%) participated in the study. Among the respondents, 500 (62.1%) were engineering students, 160 (19.9%) were science students, and 145 (18%) were liberal arts students. The participants had to sign a consent form.

### Measures

#### Basic Empathy Scale

Darrick and David (2006) compiled the BES with items that were generated based on the definitions of emotional and cognitive empathy and were drawn from four basic emotions (fear, sadness, anger, and happiness), thereby preventing social desirability bias. The scale was divided into two dimensions: emotional empathy, which comprised 11 items, and cognitive empathy, which comprised nine items, yielding a total of 20 items (including eight reverse-scoring items; Darrick and David, 2006). A five-point Likert scale was used, where 1 = “completely disagree” and 5 = “completely agree.” A high score indicated strong empathy.

#### The Gratitude Questionnaire-6

Using the Gratitude Questionnaire-6 from Mccullough et al. (2002) and the revised version by Li et al. (2012), this study adopted six items, which were measured using a seven-point Likert scale ranging from 1 = “totally disagree” to 7 = “completely agree.” Among these, a reverse-scoring item was included. After scoring, the average score of the six items was calculated. A high score indicated strong trait gratitude. The Cronbach’s α coefficient of the questionnaire in this study was 0.834.

#### The Internet Altruistic Behavior Questionnaire

This study adopted the IABQ compiled by Zheng et al. (2011). It contains 26 items and is scored on a five-point Likert scale ranging from 1 = “none” to 5 = “always.” A high score reflects an individual’s high engagement with Internet altruistic behavior. The questionnaire included four subscales: network support, network guidance, network sharing, and network reminders. In this study, the Cronbach’s α coefficient for the total scale was 0.937. The Cronbach’s α coefficients of the subscales of network support, guidance, sharing, and reminders were 0.872, 0.832, 0.766, and 0.786, respectively.

### Procedure

This study obtained authorization from Dr. Darrick Jolliffe to revise the BES. The scale was first independently translated into Chinese by a psychology professor and agreed upon after the discussion. We subsequently asked a Chinese American psychology professor to translate the Chinese-translated version back into English. We then compared the translated English with the original text, modified the items with considerable differences in translation, and further improved the accuracy of the questionnaire translation. Finally, a Chinese psychology professor and several graduate psychology students were asked to evaluate the content validity to ensure that it conformed to Chinese culture and semantics in terms of expression habits and living customs. Thirty Chinese college students were randomly selected to complete the scale since they would understand it and a final questionnaire was developed.

The questionnaire was then formally tested. First, all college students who took the test were asked to complete an informed consent form. Second, the students were asked to provide demographic data. Finally, they were asked to complete the questionnaire. The data collection process was administered by a Chinese psychology professor and several undergraduate students; the main researcher was also present in the classroom and collected the questionnaires after the students completed them. Considering that some items of the questionnaire may be traumatic and cause discomfort to the participants, we used a comforting and dignified way after the test to help the subjects get rid of the negative influence caused by the test situation. After the questionnaires were completed, they were collected by the main researcher.

After 2 months, 52 subjects were randomly selected from the sample to fill out the basic empathy questionnaire to test the reliability of our measurement.

### Data Analysis

The data were analyzed using SPSS 23.0 and Mplus 8.4. Item analysis was used to investigate the discrimination of the items. The internal consistency coefficient values were determined using reliability analysis. Evidence for construct validity was obtained through exploratory structural equation modeling (ESEM; Asparouhov and Muthén, 2009). To determine the degree of fit of the model, certain commonly used fitting indices were selected for this study: the chi-square goodness-of-fit statistic, the comparative fit index (CFI), Tucker–Lewis index (TLI), root mean square error of approximation (RMSEA), and standardized root mean square residual (SRMR). Correlation analysis was used to investigate correlations between different variables. The independent samples *t*-test was used to analyze gender differences.

## Results

### Item Analysis

We initially calculated the corrected item-total *r* (Chen et al., 2015). The corrected item-total *r* refers to the correlation coefficient between the score of each item and the total score of each item that remains in the subscale. The corrected item-total *r* of the emotional empathy subscale ranges from 0.310 to 0.514 and from 0.324 to 0.510 for the cognitive empathy subscale (Table 1). Evidently, both were greater than 0.30. Then, item-total *r* was calculated (Hao and Hong, 2014). The total *item r* refers to the correlation between the item and the total score of the corresponding subscale. The item-total *r* of the emotional empathy subscale is between 0.423 and 0.628, *ps* < 0.01, and the item-total *r* of the cognitive empathy subscale is between 0.479 and 0.657, *ps* < 0.01 (Table 1). Both values were greater than 0.30. Finally, according to the respondents’ high and low grouping at 27% before and after each subscale score with an independent sample *t-*test to compare the high and low group scores of each item, all items are significantly different. Table 1 reports the results. Therefore, all items of the scale were well discriminated.

**TABLE 1:** Corrected item–total *r*, the item–total *r*, and Cr value.

**Item number**	**Item**	**Corrected item–total *r***	**Item–total *r***	**Cr**
1	我朋友的情绪不太能影响我。 (My friends’ emotions don’t affect me much)	0.360^**^	0.506^**^	40.951^***^
2	当身边的朋友在为某事难过时,我通常会感到,难过。 (After being with a friend who is sad about something, I usually feel sad)	0.420^**^	0.541^**^	36.210^***^
4	当我看到经典的恐怖电影中的角色时,我会害怕。 (I get frightened when I watch characters in a good scary movie)	0.301^**^	0.499^**^	70.732^***^
5	我很容易陷入进别人的情感中。 (I get caught up in other people’s feelings easily)	0.421^**^	0.574^**^	58.808^***^
7	当我看到别人哭时,我不会悲伤。 (I don’t become sad when I see other people crying)	0.461^**^	0.592^**^	42.000^***^
8	别人的情感一点也不会打扰我。 (Other people’s feeling don’t bother me at all)	0.514^**^	0.628^**^	56.593^***^
11	当我在电视或电影里看悲伤的场面时,我常常,感到伤心。 (I often become sad when watching sad things on TV or in film)	0.365^**^	0.504^**^	47.225^***^
13	看到一个被激怒的人对我的情感没有任何影响。 (Seeing a person who has been angered has no effect on my feelings)	0.362^**^	0.506^**^	37.533^***^
15	当我和害怕的朋友在一起时,我容易感到害怕。 (I tend to feel scared when I am with friends who are afraid)	0.310^**^	0.433^**^	60.515^***^
17	我经常卷进朋友的情感中。 (I often get swept up in my friends’ feelings)	0.312^**^	0.456^**^	50.930^***^
18	我朋友的不幸并没有让我有任何感觉。 (My friend’s unhappiness doesn’t make me feel anything)	0.323^**^	0.453^**^	35.809^***^
3	当我的朋友在某事上做得很好时,我能理解他/她的快乐。 (I can understand my friend’s happiness when she/he does well at something)	0.324^**^	0.479^**^	27.544^***^
6	我发现我很难意识到我的朋友在经受恐惧。 (I find it hard to know when my friends are frightened)	0.331^**^	0.525^**^	39.854^**^
9	当有人感到“失落”时,我通常能理解他们的感受。 (When someone is feeling “down,” I can usually understand how they feel)	0.341^**^	0.501^**^	34.066^***^
10	当我的朋友害怕的时候,我通常都能够感受到。 (I can usually work out when my friends are scared)	0.423^**^	0.569^**^	33.270^***^
12	我能够在人们告诉我之前就理解他们的感受。 (I can often understand how people are feeling even before they tell me)	0.366^**^	0.541^**^	34.106^***^
14	我通常能看出人们什么时候高兴。 (I can usually work out when people are cheerful)	0.386^**^	0.544^**^	35.456^***^
16	当我朋友生气,我可以很快意识到。 (I can usually realize quickly when a friend is angry)	0.402^**^	0.557^**^	37.687^***^
19	我通常不能意识到我朋友们的感受。 (I am not usually aware of my friends’ feelings)	0.510^**^	0.657^**^	50.425^***^
20	我很难弄清楚我的朋友们什么时候开心。 (I have trouble figuring out when my friends are happy)	0.467^**^	0.625^**^	48.426^***^

**p < 0.01, ***p < 0.001, emotional empathy: item 1, 2, 4, 5, 7, 8, 11, 13, 15, 17, 18. cognitive empathy: item 3, 6, 9, 10, 12, 14, 16, 19, 20.

### Validity Analysis

#### Exploratory Structural Equation Modeling

After the reverse score of the relevant items, a two-factor model of the original data was conducted. The questionnaire’s initial design theory set a two-factor model that included two related factors: emotional empathy (Factor I) and cognitive empathy (Factor II). Table 2 presents the fitting indices of ESEM. The two-factor fitting indices illustrate that the two-factor model failed to fit the data well.

**TABLE 2:** Goodness-of-fit and indices for the competitive models of the BES.

**Model**	**χ^2^**	***df***	**CFI**	**TLI**	**RMSEA**	**SRMR**
Two factors	805.904^***^	151	0.775	0.716	0.073	0.055
Two factors with methodological effect	409.648^***^	133	0.868	0.812	0.051	0.038
Correction two factors with methodological effect	249.767 ^***^	128	0.942	0.933	0.034	0.030

***p < 0.001. CFI, the comparative fit index; TLI, the Tucker Lewis Index; RMSEA, the root-mean-square error of approximation; SRMR, the standardized root mean square residual.

**FIGURE 1:**
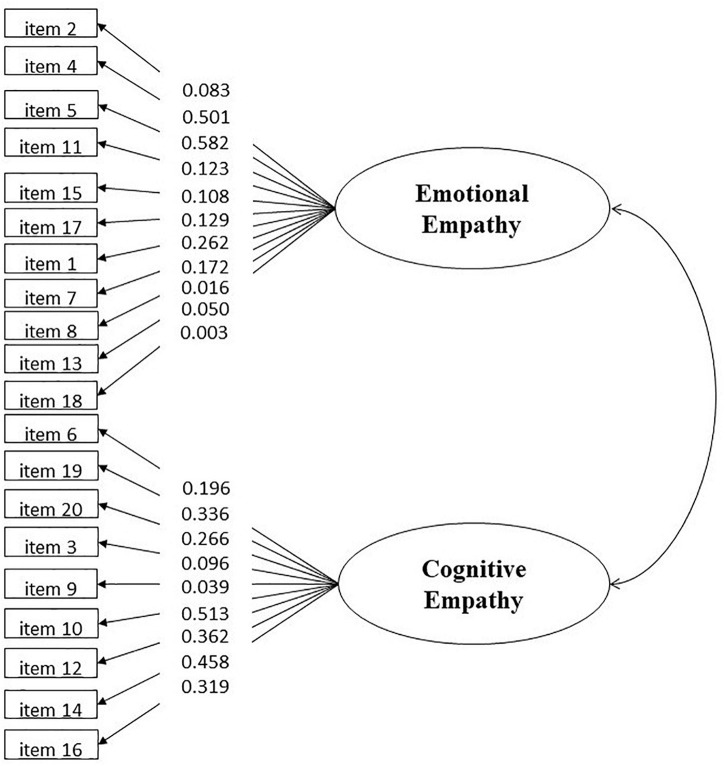
Two factors model with methodological effect.

Combined with Li et al.’s (2011) study on the analysis of the two-factor model of the revised empathy scale for adolescents, the two-factor model with the methodological effect was further examined in this study. Based on the aforementioned emotional and cognitive empathy dimensions, a methodological effect dimension was added, and its loading was derived from all reverse-scored items (Figure 1). Table 2 lists the fitting indices. Although the CFI and TLI were not within the acceptable cut-off (0.90), RMSEA and SRMR met the psychometric requirements (Hai and Wen, 2013). Some item residuals are strongly correlated with each other. In the correction of the covariant relation of the item residuals, the correlation between two item residuals with the largest MI index was gradually established. Subsequently, the correction model fitting indices improved the desirability. Table 2 shows that all indices were accepted.

Based on the three-factor model found in the revised basic empathy scale among the adult population by Carré et al. (2013), the fit of the model among Chinese college students was examined. However, no convergence was observed. The most common reason for model non-convergence is that latent variables cannot be identified. The failure to identify latent variables is mainly reflected in the collinearity between indicators, insignificant loading, and too few measurement indicators. Therefore, the model was rejected.

#### Correlation and Convergent Validity

In this study, the GQ-6 and IABQ served as questionnaires to test the convergent validity of the BES. Table 3 illustrates the correlation matrix of the BES, the GQ-6 and IABQ. Correlations between the two scales of BES were 0.304 (*p* < 0.01) for the total sample. The BES total scores positively correlated significantly with the two subscales. The analysis results also revealed a significant positive correlation between empathy, gratitude and Internet altruistic behavior.

**TABLE 3:** Correlations among scales of the BES, GQ-6, and the IABQ (*N* = 805).

**Scale**	**1**	**2**	**3**	**4**	**5**	**6**	**7**	**8**	**9**
1.Empathy	1								
2. Emotional empathy	0.828^**^	1							
3. Cognitive empathy	0.786^**^	0.304^**^	1						
4. Trait gratitude	0.371^**^	0.242^**^	0.364^**^	1					
5. Internet altruistic behavior	0.164^**^	0.124^**^	0.142^**^	0.139^**^	1				
6. Network support	0.226^**^	0.192^**^	0.172^**^	0.167^**^	0.874^**^	1			
7. Network guidance	0.065	0.023	0.084^*^	0.044	0.871^**^	0.652^**^	1		
8. Network sharing	0.059	0.028	0.070^*^	0.072^*^	0.852^**^	0.657^**^	0.719^**^	1	
9. Network reminders	0.203^**^	0.170^**^	0.158^**^	0.187^**^	0.871^**^	0.709^**^	0.659^**^	0.624^**^	1

*p < 0.05, **p < 0.01.

### Reliability Analysis

The reliability analysis revealed that the Cronbach’s α coefficients of the emotional and cognitive empathy subscales were the same at 0.72. Furthermore, the Cronbach’s α coefficient of the total scale was 0.767. The test-retest correlations of the emotional and cognitive empathy subscales were statistically significant at 0.853 and 0.831, respectively, indicating acceptable temporal stability.

### Gender Differences

The data were tested for gender differences. The mean score of the boys’ emotional empathy dimension was 3.38 (*SD* ± 0.52), and their mean cognitive empathy dimension score was 3.66 (*SD* ± 0.47); the mean score of the girls’ emotional empathy dimension was 3.63 (*SD* ± 0.48), and their mean cognitive empathy dimension score was 3.73 (*SD* ± 0.46). Thus, girls scored significantly higher than boys in the emotional empathy dimension (*t* = 6.622, *p* < 0.001), and the cognitive empathy dimension did not differ significantly (*t* = 1.902, *p* = 0.057).

## Discussion

This study revised the BES for suitability for college students in China. Item analysis revealed that the 20 items in the questionnaire exhibited good item discrimination. The internal consistency coefficients of the emotional and cognitive empathy subscales were the same at 0.72. Additionally, the reliability of the total scale was 0.767. The test-retest correlations were 0.853 and 0.831, respectively. Therefore, the psychometric standards were satisfied. The factor analysis results revealed that the internal structure and number of items in the revised questionnaire were similar to those in the original questionnaire. The correction model fit indices revealed that the CFI and TFI were greater than 0.90, and the RMSEA and SRMR were less than 0.08. Thus, all fit indices satisfied the psychological measurement standards, and the scale exhibited a clear structure.

In this study, the GQ-6 and IABS were used as questionnaires to test the convergent validity of the BES. The results showed that cognitive empathy, emotional empathy, and gratitude were significantly and positively correlated with the BES scale. Gratitude was fundamentally triggered by the perception of life experiences and positive recognition of the beneficial activities of others (Liu and Liang, 2011). In the dynamic model of empathy, cognition is an important link, and high gratitude improves individual physical recognition of other people’s behavior. Thus, this factor has a high empathy ability. Moreover, individuals with high gratitude tend to have strong sympathetic reactions to others’ negative experiences (Mccullough et al., 2002) and can feel and sense other people’s emotions, thereby increasing their empathy toward others. Furthermore, the findings indicate that empathy is significantly and positively related to altruism. Notably, Batson’s empathy–altruism theory suggests that empathy is the key to and an important source of altruism (Batson, 1987). Furthermore, intense empathy indicates a strong level of altruism to help alleviate others’ difficulties. In the network environment, people with high levels of empathy are aware of a person’s difficult state and are likely to exhibit Internet altruistic behavior, which is consistent with previous studies (Zheng and Li, 2006; Zheng and Zhao, 2015; Jiang et al., 2016).

The results of the gender difference test revealed that girls performed better than boys on the emotional empathy subscale, which was consistent with previous studies. Research has found that girls have an advantage in empathy responses (Preti et al., 2011; O’Brien et al., 2013). According to the theory of mirror neurons, cognitive neuroscience studies have found gender differences in the mirror nervous system of humans, with females using the mirror nervous system more frequently than males in perceiving others (Cheng et al., 2008); hence, they are more likely to produce experiences similar to others. Thus, females had a higher level of empathy than males. In terms of psychological characteristics, females have higher interpersonal sensitivity, particularly toward negative emotional events, than males. Thus, women process social information and experience other people’s emotions more intensely than males, which is one of the reasons for the gender difference in emotional empathy at the psychological level (Su, 2014). The socialization of individual gender roles is another reason for the gender difference in empathy (Chen et al., 2014). The gender role socialization theory posits that social culture and education expect women to pay attention to other people’s emotions, given that they are likely to provide empathetic responses to the difficulties of others. Meanwhile, men are expected to be more independent than women. Thus, they tend to solve problems through rational thinking and are less likely to display emotionally empathetic responses toward the difficulties of others.

In conclusion, the revised version of the BES exhibits good reliability and validity and can be used as a tool to evaluate college Students’ empathy ability in China. Moreover, empathy can predict internet altruism (Jiang et al., 2016). Thus, the questionnaire can also predict college Students’ internet altruism from an empathy perspective.

## Limitations and Future Directions

First, this study selected university students as participants, but non-clinical samples were used. In the future, clinical samples from college students could be selected to expand the applicability of the scale. Second, a self-report method was adopted in this study. These results may have been affected by the social approval effect. In the future, other methods, such as interviews, can be used to further verify the reliability and validity of the scale. Third, as the measurement invariance test was not carried out in this study, it is unknown whether there is the same factor structure in different groups. Therefore, a measurement invariance test would be used to investigate the structural consistency of empathy in different groups in the future.

## Data Availability Statement

The original contributions presented in the study are included in the article/supplementary material, further inquiries can be directed to the corresponding author/s.

## Ethics Statement

The studies involving human participants were reviewed and approved by the Research Ethics Committee of the University of Science and Technology Liaoning (China). The patients/participants provided their written informed consent to participate in this study.

## Author Contributions

YC and LC reviewed the literature and wrote the manuscript. YC and GD outlined the structure of the manuscript, reviewed the literature, and wrote the manuscript. All authors contributed to the article and approved the submitted version.

## Conflict of Interest

The authors declare that the research was conducted in the absence of any commercial or financial relationships that could be construed as a potential conflict of interest.

## Publisher’s Note

All claims expressed in this article are solely those of the authors and do not necessarily represent those of their affiliated organizations, or those of the publisher, the editors and the reviewers. Any product that may be evaluated in this article, or claim that may be made by its manufacturer, is not guaranteed or endorsed by the publisher.
